# Dynamics of growth and weight transitions in a pediatric cohort from India

**DOI:** 10.1186/1475-2891-8-55

**Published:** 2009-11-23

**Authors:** Manu Raj, Karimassery R Sundaram, Mary Paul, Abish Sudhakar, Raman K Kumar

**Affiliations:** 1 Department of Pediatric Cardiology, Amrita Institute of Medical Sciences and Research Centre, Kochi, Kerala, India; 2 Department of Biostatistics, Amrita Institute of Medical Sciences and Research Centre, Kochi, Kerala, India

## Abstract

**Background:**

There is paucity of information regarding time trends of weight status in children from rapidly developing economies like India. The aim of the study was to analyse the dynamics of growth and weight transitions in a cohort of school children from India.

**Methods:**

A population of 25 228 children was selected using stratified random sampling method from schools in a contiguous area in Ernakulam District, Kerala, India. Weight and height were measured at two time points, one in 2003-04 and another in 2005-06. The paired data of 12 129 children aged 5-16 years were analysed for the study.

**Results:**

The mean interval between the two surveys was 2.02 ± 0.32 years. The percentage of underweight, normal weight, overweight and obese children in the year 2003-04 were 38.4%, 56.6%, 3.7%, and 1.3% respectively. The corresponding figures in year 2005-06 were 29.9%, 63.6%, 4.8% and 1.7% respectively. Among the underweight children, 34.8% migrated to normal weight status and 0.1% migrated to overweight status. Conversion of underweight to normal weight predominated in urban area and girls. Among the normal weight children, 8.6% migrated to underweight, 4.1% migrated to overweight and 0.4% migrated to obesity. Conversion of normal weight to overweight status predominated in urban area, private schools and boys. Conversion of normal weight to underweight predominated in rural area, government schools and boys. Among the overweight children, 26.7% migrated to normal weight status, 16.4% became obese and 56.9% retained their overweight status. Of the obese children, 6.2% improved to normal weight status, 25.3% improved to overweight status and 68.5% remained as obese in 2005-06. There was significant difference in trends between socio demographic subgroups regarding conversion of underweight status to normal weight as well as normal weight status to overweight.

**Conclusion:**

The study population is experiencing rapid growth and nutritional transitions characterised by a decline in the underweight population coupled with an escalation of the overweight population. The heterogeneous nature of this transition appears to be due to differences in socio demographic factors.

## Background

Globally, countries are passing through concurrent epidemiological, demographic and nutritional transitions [1]. The term nutritional transition is used to characterize the shift in disease patterns towards nutrition related non communicable diseases. This shift in disease patterns is associated with changes in behaviour, lifestyles, diets, physical activity, smoking and alcohol consumption [2]. The rapidity of such nutritional transitions is clearly visible in emerging nations from Asia. Countries like India, China, Thailand, Indonesia and South Korea are currently witnessing such transitions [3-7]. There is evidence of such a rapid nutritional transition amplifying the burden of chronic diseases and obesity in India [3]. Economic reforms have modified pediatric growth patterns as well as prevalence of under nutrition and overweight in large economies like China [4]. Time trends in childhood obesity reported from India predicts an escalating burden of obesity related issues in the near future [8,9]. In spite of these rapid changes, many countries have significant levels of malnutrition and child mortality together with rising prevalence of obesity and non communicable diseases [1]. This double burden is a result of an interaction between various factors of which social inequality merits more attention than others. Social inequality has emerged as a major factor in differential mortality in both developed and developing countries [1].

The relationship between socioeconomic levels and weight status shows interesting asymmetry [10]. The urban poor of developed economies are particularly vulnerable to childhood obesity in contrast to developing nations where the urban rich are extremely at risk for the same [11]. While the former group appears vulnerable due to poor diet and decreasing physical activity, the latter remains at risk probably due to an increased affinity towards a western type of lifestyle [12-15]. A recent systematic review demonstrated that associations between physical environmental variables and obesity differ by gender, age, socio economic status and population density [16]. Cohort studies have demonstrated that social patterning of overweight varies between and within populations over time [17]. National surveys from China have demonstrated the rapidity of overweight progression as well as the differential growth of overweight in terms of varying levels of urbanization [18]. In Thailand, the prevalence of overweight and obesity among children and adolescents increased dramatically during the last two decades [5]. This was more pronounced in children from private schools and urban communities than in those from public schools or rural communities. Studies from India have also stressed the role of socio economic status and urbanization in promoting childhood obesity [8,19]. The aim of this study was to examine the dynamics of growth and weight transitions in school children from a selected population in India and to assess the influence of socio demographic factors in modifying these transitions.

## Methods

A contiguous area with a population of approximately 1.37 million was selected from Ernakulam district, in central Kerala, South India. Sampling was done by stratified random sampling method. Schools in the area were stratified into 5 groups according to the number of students and a representative sample of 46 schools with a cumulative population of 25 228 children was randomly chosen. Anthropometric measurements, which consisted of height and weight were recorded for 24 842 children of age group 5-16 years in 2003-04. The same set of measurements was repeated in the selected schools in 2005-06 covering a total of 20 263. The anthropometric measurements were recorded by personnel specifically trained for the study. One trained person was dedicated for recording weight and another for height, to avoid inter observer error. Intra observer error was within acceptable limits for both height and weight measurements as documented by the co-efficient of reliability (R > 0.99). Height was measured to the nearest 0.5 centimeter by a wall-mounted stadiometer. Weight was measured to the nearest 0.5 kilogram by a mechanical weighing scale. Both equipments were standardized at regular intervals. A total of 12 129 children had two sets of measurements, one in 2003-04 and other in 2005-06. Paired data of these children were used for studying dynamics of growth and weight trends in the study population. Children with Body Mass Index (BMI) less than or equal to 5^th ^percentile of reference data were considered underweight. Children with BMI more than or equal to 85^th ^and less than 95^th ^percentile of reference data were considered overweight. Children having BMI more than or equal to 95^th ^percentile were considered obese [20]. The reference data used to identify the BMI cut offs as well as conversion of weight and height to Z scores were taken from CDC 2000 data set for growth parameters in children and adolescents [21]. Age in months was used for converting BMI, weight and height to Z scores as per CDC reference. The cohort was divided into various subgroups for detailed analysis. Schools were divided into government and private schools. Government schools receive subsidies from the educational department enabling them to provide education at less than INR 500 per year per student (approximately US$ 12). Private schools receive no subsidies and charge students INR 5000 and above per year. Schools were also divided into rural and urban as well. Rural area was defined if more than 75 percent of adult male population was engaged in agricultural occupations along with lower levels of developmental indices. Non-rural areas were designated as urban areas.

### Statistical analysis

The data were analyzed using SPSS software version 15. Anthropometric transition was assessed by converting the corresponding parameters to Z scores and comparing their means. Paired samples test was used for comparing individual subgroup time transitions. Independent samples test was used for comparing time transitions of inter subgroup differences. Pearson Chi square test was used for comparing weight transitions among various sub groups. Significance was assigned for a P value < 0.05.

### Ethical approval

Approval for the study was obtained from the ethical committee of the home institution in compliance with the guidelines issued by Indian Council of Medical Research. Consent to conduct the survey on students was obtained from parents through school authorities, who arranged parent meetings in the respective schools. Verbal assent was taken from the children after demonstrating and explaining the procedure.

## Results

Descriptive data of the cohort based on the two periods 2003-04 and 2005-06 is given in Table 1. Age and gender specific BMI percentiles of the cohort with respect to socio demographic subgroups are given in Table 2. The mean interval between the two surveys was 2.02 ± 0.32 years. The percentage of underweight, normal weight, overweight and obese children in the year 2003 were 38.4%, 56.6%, 3.7%, and 1.3% respectively. The corresponding figures when the cohort was examined two years later were 29.9%, 63.6%, 4.8% and 1.7% respectively. The difference in categories of weight status between 2003 and 2005 appears to be statistically significant (P < 0.0001).

**Table 1:** Descriptive data of the study cohort

2003	Boys				Girls			
**Age** **(yrs)**	**N**	**Height ** **(cm)**	**Weight** **(kg)**	**BMI** **(kg/m^2^)**	**N** **(cm)**	**Height** **(kg)**	**Weight** **(kg/m^2^)**	**BMI**

5	293	109.7 (4.6)	16.7 (2.2)	13.8 (1.3)	223	108.6 (5.5)	16.3 (2.4)	13.8 (1.5)
6	580	115.5 (5.4)	19.1 (3.2)	14.2 (1.6)	486	114.3 (5.5)	18.6 (3.5)	14.2 (1.8)
7	574	120.7 (6.2)	21.1 (4.1)	14.4 (1.9)	425	120.2 (5.8)	20.8 (3.8)	14.3 (1.8)
8	534	126.6 (6.1)	23.8 (4.6)	14.8 (2.0)	421	125.5 (6.4)	23.4 (5.3)	14.7 (2.2)
9	720	131.9 (6.5)	26.4 (6.0)	15.1 (2.3)	737	131.4 (6.5)	26.4 (5.6)	15.1 (2.3)
10	812	135.9 (6.8)	28.5 (6.9)	15.3 (2.6)	946	136.5 (7.1)	28.8 (6.2)	15.3 (2.3)
11	733	140.5 (7.4)	31.2 (7.5)	15.6 (2.7)	1059	142.3 (7.1)	32.7 (7.1)	16.0 (2.6)
12	842	145.9 (7.4)	33.9 (7.4)	15.8 (2.5)	1122	147.9 (7.2)	36.7 (8.1)	16.6 (2.8)
13	556	152.5 (8.7)	39.2 (9.8)	16.7 (3.0)	706	151.0 (6.7)	39.7 (7.7)	17.3 (2.7)
14	197	155.9 (8.7)	41.6 (9.7)	17.0 (2.9)	146	151.9 (6.1)	40.9 (7.3)	17.7 (2.7)
**2005**								
7	207	122.4 (5.1)	21.3 (3.8)	14.1 (1.8)	163	122.1 (5.7)	21.6 (5.1)	14.4 (2.4)
8	592	127.4 (5.8)	24.5 (5.2)	15.0 (2.3)	489	126.1 (6.3)	23.7 (4.9)	14.8 (2.1)
9	574	131.8 (6.5)	26.8 (5.9)	15.3 (2.4)	454	131.7 (6.6)	26.6 (5.7)	15.2 (2.3)
10	551	137.5 (6.8)	30.1 (6.8)	15.8 (2.6)	406	138.0 (7.2)	30.6 (7.4)	15.9 (2.7)
11	746	142.5 (7.5)	33.3 (8.1)	16.2 (2.8)	763	143.7 (7.4)	34.4 (8.2)	16.5 (2.8)
12	837	146.7 (8.0)	35.9 (9.2)	16.5 (3.0)	989	148.7 (6.6)	38.4 (8.3)	17.2 (2.9)
13	754	153.7 (8.7)	40.7 (9.4)	17.1 (2.9)	1034	152.0 (6.0)	41.4 (7.5)	17.9 (2.8)
14	836	160.1 (8.2)	45.0 (9.4)	17.4 (2.7)	1204	154.5 (5.8)	44.1 (8.6)	18.4 (3.1)
15	537	164.3 (7.4)	49.3 (10.0)	18.2 (2.9)	648	155.2 (6.3)	45.9 (8.4)	19.0 (2.9)
16	211	166.1 (6.7)	52.7 (10.7)	19.0 (3.2)	131	155.3 (6.4)	46.2 (8.4)	19.1 (3.1)

Mean values of height, weight and body mass index. Figures in parenthesis are standard deviations.

Data of children in age group 15 years in 2003 (n = 6) and age group 6 years in 2005 (n = 2) are not shown in the table, as the number of children in the same is very low.

**Table 2:** Age and gender specific mean BMI values according to socio demographic subgroups

2003	Boys				Girls			
**Age (yrs)**	**Urban**	**Rural**	**Govt **	**Private**	**Urban **	**Rural **	**Govt **	**Private**

5	13.7 (1.2)	13.9 (1.4)	13.8 (1.3)	14.2 (1.4)	13.7 (1.4)	13.8 (1.5)	13.8 (1.4)	14.5 (1.6)
6	14.5 (1.6)	13.9 (1.5)	13.9 (1.4)	15.0 (1.7)	14.5 (2.1)	13.9 (1.4)	13.8 (1.4)	15.2 (2.4)
7	14.6 (2.1)	13.9 (1.4)	13.9 (1.5)	15.3 (2.2)	14.7 (2.0)	13.9 (1.5)	13.9 (1.5)	15.1 (2.1)
8	15.1 (2.2)	14.2 (1.4)	14.3 (1.4)	15.5 (2.4)	15.2 (2.5)	14.3 (1.7)	14.3 (1.8)	15.5 (2.6)
9	15.6 (2.6)	14.5 (1.9)	14.5 (1.9)	16.3 (2.7)	15.5 (2.5)	14.8 (2.0)	14.8 (2.1)	16.3 (2.7)
10	15.8 (2.9)	14.6 (2.1)	14.7 (2.1)	16.9 (3.2)	15.6 (2.5)	15.0 (2.1)	15.1 (2.2)	16.8 (2.7)
11	16.2 (3.0)	14.9 (2.2)	15.0 (2.2)	17.2 (3.2)	16.3 (2.7)	15.6 (2.3)	15.8 (2.5)	17.1 (3.0)
12	16.1 (2.7)	15.5 (2.2)	15.4 (2.2)	17.4 (2.7)	16.9 (2.9)	16.2 (2.6)	16.4 (2.7)	18.2 (3.3)
13	17.2 (3.3)	15.8 (2.0)	16.0 (2.4)	18.2 (3.6)	17.5 (2.7)	16.8 (2.5)	17.1 (2.6)	18.2 (2.7)
14	17.1 (3.3)	16.8 (2.5)	16.8 (2.7)	18.4 (3.8)	17.8 (2.7)	17.2 (2.7)	17.6 (2.7)	18.4 (2.8)
**2005**								
7	14.4 (2.0)	14.0 (1.5)	13.9 (1.4)	15.6 (2.8)	14.6 (3.1)	14.2 (1.7)	14.0 (1.6)	16.1 (4.2)
8	15.5 (2.4)	14.3 (1.9)	14.5 (2.0)	16.1 (2.6)	15.0 (2.3)	14.5 (1.8)	14.4 (1.7)	15.8 (2.5)
9	15.8 (2.5)	14.5 (2.0)	14.6 (1.9)	16.4 (2.7)	15.9 (2.6)	14.5 (1.6)	14.6 (1.7)	16.4 (2.7)
10	16.3 (2.8)	14.8 (1.9)	15.1 (2.1)	16.8 (2.9)	16.2 (2.8)	15.5 (2.6)	15.4 (2.5)	16.8 (2.9)
11	16.8 (2.9)	15.6 (2.5)	15.7 (2.4)	17.5 (3.1)	16.8 (3.0)	16.1 (2.4)	16.2 (2.6)	17.7 (3.2)
12	17.0 (3.2)	15.9 (2.6)	15.9 (2.5)	18.3 (3.7)	17.6 (3.1)	16.8 (2.6)	17.0 (2.8)	18.6 (3.1)
13	17.5 (3.1)	16.5 (2.5)	16.6 (2.6)	18.4 (3.3)	18.1 (2.9)	17.5 (2.6)	17.8 (2.7)	18.7 (2.9)
14	17.7 (2.9)	17.2 (2.5)	17.1 (2.4)	18.8 (3.3)	18.6 (3.2)	18.0 (2.9)	18.2 (3.0)	20.1 (3.6)
15	18.5 (3.3)	17.7 (2.1)	17.7 (2.5)	19.4 (3.5)	19.2 (2.9)	18.5 (2.7)	18.8 (2.8)	19.7 (3.0)
16	19.3 (3.6)	18.7 (2.6)	18.7 (2.8)	20.7 (4.2)	19.2 (2.9)	19.0 (3.6)	19.0 (3.1)	20.3 (2.6)

Figures in parenthesis are standard deviations

Govt- government schools

Among the 4658 underweight children in 2003-04, 65.2% remained underweight and the rest migrated to other weight groups. Of the 6864 normal weight children in 2003-04, 86.9% remained normal and the rest migrated to other weight groups. Among the 445 overweight children in 2003-04, 56.9% retained their overweight status and the remaining migrated to other weight groups. Of the 162 obese children in 2003-04 68.5% remained as obese and the rest migrated to other weight groups in 2005-06. No child who was overweight or obese in 2003-04 migrated to underweight status in 2005-06. Similarly, no child who was under weight in 2003-04 became obese in 2005-06 screening. The details of the weight transitions across various weight groups are presented in Table 3.

**Table 3:** Weight transitions across weight groups (2003 to 2005)

Weight status 2003	Total	Weight status 2005			
		**UW**	**NW**	**OW**	**OB**
**UW**	4658	3035 (65.2)	1619 (34.8)	4 (0.1)	0
**NW**	6864	590 (8.6)	5963 (86.9)	284 (4.1)	27 (0.4)
**OW**	445	0	119 (26.7)	253 (56.9)	73 (16.4)
**OB**	162	0	10 (6.2)	41 (25.3)	111 (68.5)

Figures in parenthesis are percentages

UW -- Underweight, NW- Normal weight, OW- Overweight (excluding obese), OB- Obese

**Figure 1:**
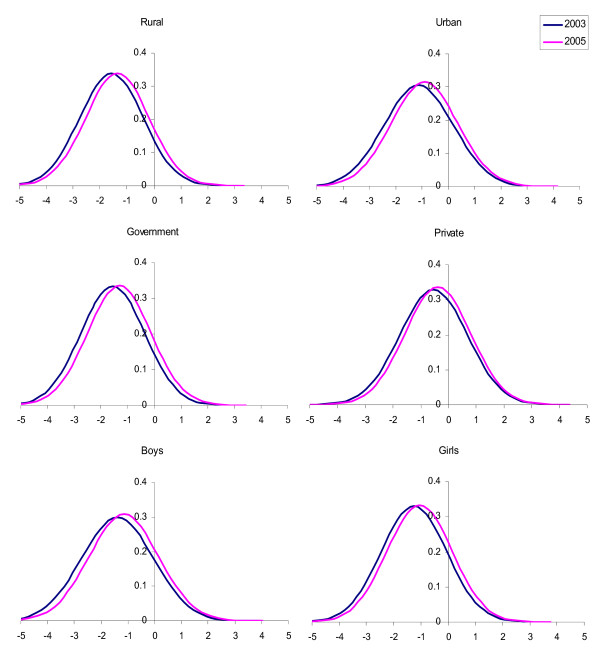
**Normal distribution curves for weight Z scores of subgroups**. The curve labeled 2003 corresponds to distribution at the start of the study and the one labeled 2005 corresponds to distribution at the end of the study.

**Figure 2:**
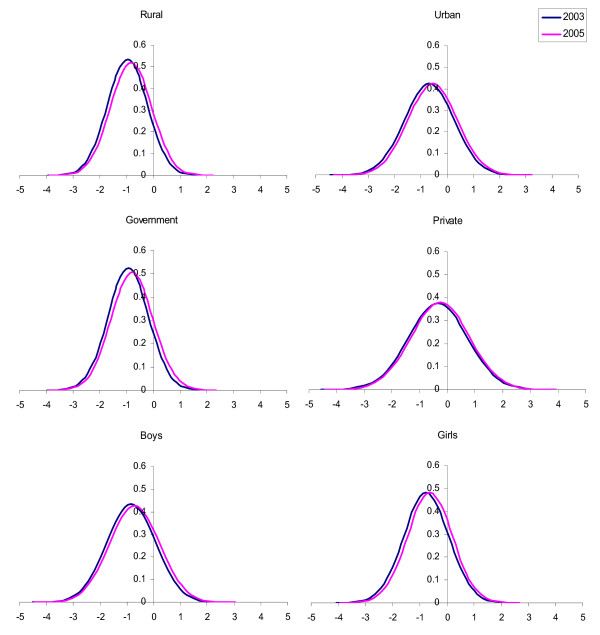
**Normal distribution curves for BMI Z scores of subgroups**. The curve labeled 2003 corresponds to distribution at the start of the study and the one labeled 2005 corresponds to distribution at the end of the study.

**Figure 3:**
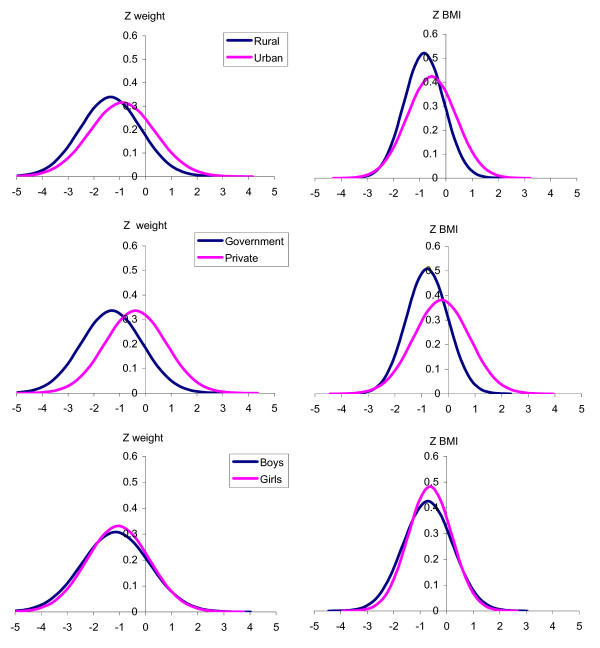
**Comparison of final normal distribution curves of weight and BMI Z scores between subgroups**. The normal distribution curves of weight and BMI Z scores were constructed from observations at the end of the study.

The anthropometric parameters (height, weight and BMI) were converted to their respective Z scores based on reference population values [21] and analyzed (Table 4). The cohort showed significant improvements in height, weight and BMI during the study period as documented by corresponding increases in their mean Z scores (P < 0.001). Sub-group analysis of the cohort was done to look for the influence of socio demographic factors in this anthropometric transition. The rural subgroup showed significant improvements in weight and BMI Z scores (P < 0.001). The change in height Z score was not significant. The urban sub group showed significant improvements in all three parameters (P < 0.001). The government school subgroup showed significant improvements in weight and BMI Z scores (P < 0.001) only where as private school group showed significant improvements in all three parameters (P < 0.001). The boys sub group demonstrated an increase in all three parameters (P < 0.001) where as girls demonstrated an increase in weight and BMI Z scores only (P < 0.001). In addition girls demonstrated a significant reduction in height Z score (P < 0.01). The transitions of weight and BMI Z scores across various sub groups are presented as Figures 1 and 2. The sub group based segregation of weight and BMI Z score distribution at the end of the study is presented as Figure 3.

**Table 4:** Trends in Z scores of Anthropometric parameters

Group		Mean Z-height			Mean Z-weight			Mean Z-BMI		
	**N**	**2003**	**2005"**	sig*	**2003**	**2005**	sig*	**2003**	**2005**	sig*

**All**	12129	-0.81 (1.02)	-0.79 (0.99)	< 0.001	-1.31 (1.27)	-1.09 (1.25)	< 0.001	-0.80 (0.88)	-0.67 (0.88)	< 0.001
**Rural**	5240	-0.94 (0.99)	-0.94 (0.95)	0.823	-1.57 (1.18)	-1.36 (1.18)	< 0.001	-0.97 (0.75)	-0.83 (0.77)	< 0.001
**Urban**	6889	-0.72 (1.03)	-0.68 (1.01)	< 0.001	-1.12 (1.31)	-0.90 (1.26)	< 0.001	-0.67 (0.94)	-0.54 (0.94)	< 0.001
sig**		**< 0.001**			**0.416**					**0.887**
Govt	9358	-0.96 (0.98)	-0.96 (0.94)	0.488	-1.54 (1.20)	-1.31 (1.19)	< 0.001	-0.94 (0.76)	-0.79 (0.78)	< 0.001
**Pvt**	2771	-0.31 (0.96)	-0.24 (0.95)	< 0.001	-0.54 (1.22)	-0.38 (1.19)	< 0.001	-0.33 (1.06)	-0.25 (1.05)	< 0.001
sig**		**< 0.001**			**< 0.001**					**< 0.001**
Boys	5847	-0.79 (1.02)	-0.74 (1.02)	< 0.001	-1.38 (1.34)	-1.15 (1.29)	< 0.001	-0.84 (0.92)	-0.71 (0.94)	< 0.001
**Girls**	6282	-0.83 (1.01)	-0.85 (0.97)	< 0.008	-1.25 (1.21)	-1.05 (1.20)	< 0.001	-0.76 (0.83)	-0.63 (0.83)	< 0.001
sig**		**< 0.001**			**0.008**			**0.602**		

Figures in parenthesis are standard deviations.

* Significance of paired sample test value

**Significance of independent sample test value

Govt- government schools, Pvt- private schools

The inter subgroup differences between the two time points were analyzed to look for any significant trends (Table 4). While the rural urban difference for height increased significantly during the study period (P < 0.001), the same for weight and BMI showed no significant changes. In terms of government private comparison, the difference in height increased significantly (P < 0.001), while that of weight (P < 0.001) and BMI decreased significantly (P < 0.001). In comparing the gender subgroups, significant increase was found in height difference (P < 0.001) where as weight difference showed a significant decrease (P < 0.01). There was no significant change for difference in BMI.

For the sake of comparing demographic influences on the transition of weight status, overweight and obesity were grouped together to form the overweight group. The details are available in Table 5.

**Table 5:** Trends in weight status by socio demographic factors

2003 status					2005 status			
		**Total**	**UW**	**P value**	**Normal **	**P value**	**OW**	**P value**
**UW**	Urban	2289	1435 (62.7)	< 0.001	852 (37.2)	< 0.001	2 (0.1)	0.67
	Rural	2369	1600 (67.5)		767 (32.4)		2 (0.1)	
**NW**	Urban	4123	313 (7.6)	< 0.001	3595 (87.2)	0.335	215 (5.2)	< 0.001
	Rural	2741	277 (10.1)		2368 (86.4)		96 (3.5)	
**OW**	Urban	477	0	-	96 (20.1)	0.194	381 (79.9)	0.194
	Rural	130	0		33 (25.4)		97 (74.6)	
**UW**	Govt	4078	2654 (65.1)	0.773	1420 (34.8)	0.809	4 (0.1)	0.587
	Private	580	381 (65.7)		199 (34.3)		0 (0.0)	
**NW**	Govt	5015	466 (9.3)	< 0.001	4357 (86.9)	0.980	192 (3.8)	< 0.001
	Private	1849	124 (6.7)		1606 (86.9)		119 (6.4)	
**OW**	Govt	265	0	-	65 (24.5)	0.082	200 (75.5)	0.082
	Private	342	0		64 (18.7)		278 (81.3)	
**UW**	Boys	2573	1797 (69.8)	< 0.001	775 (30.1)	< 0.001	1 (0.04)	0.240
	Girls	2085	1238 (59.4)		844 (40.5)		3 (0.1)	
**NW**	Boys	2945	332 (11.3)	< 0.001	2445 (83.0)	< 0.001	168 (5.7)	< 0.001
	Girls	3919	258 (6.6)		3518 (89.8)		143 (3.6)	
**OW**	Boys	329	0	-	70 (21.3)	0.987	259 (78.7)	0.987
	Girls	278	0		59 (21.2)		219 (78.8)	

Figures in parenthesis are percentages

UW -- Underweight

NW- Normal weight

OW- Overweight (inclusive of overweight and obesity)

Govt-Government schools

Among children who were underweight in the first survey, more from rural area remained underweight in the second survey than those from urban area (67.5% Vs 62.7%, P < 0.001). Among those who were underweight in the first survey, more children from urban area migrated to normal weight status in the second survey than from rural area (37.2% Vs 32.4%, P < 0.001). The persistence of underweight during both surveys was more among boys compared to girls (69.8% Vs 59.4%, P < 0.001). The conversion of underweight status in the first survey to normal weight status in the second survey was seen more among girls than boys (40.5% Vs 30.1%, P < 0.001).

Among the normal weight children in the first survey, more from the rural area became underweight in the second survey compared to those from urban area (10.1% Vs 7.6%, P < 0.001).

Of the children with normal weight status in the first survey, more children from government schools became underweight in the second survey compared to those from private schools (9.3% Vs 6.7%, P < 0.001). Among the children with normal weight status in the first survey, more boys converted to under weight status in the second survey compared to girls (11.3% Vs 6.6%, P < 0.001). Among the children with normal weight status in the first survey, more boys migrated to overweight status in the second survey compared to girls (5.7% Vs 3.6%, P < 0.001). Among the children with normal weight status in the first survey, more girls retained their normal weight status in the second survey when compared to boys (89.8% Vs 83%, P < 0.001). No significant difference was seen between sub groups for transition of overweight status in the first survey to normal weight status in the second survey. Similarly no significant difference was noted between subgroups for persistence of overweight status in the two surveys.

## Discussion

The dynamics of growth transition demonstrated by the cohort appears to be heterogeneous in nature. The positive shift in weight appears to be more when compared to that seen in height. A similar trend was reported by Vidal et al [22]. The rural as well as government school children have shown significant increments in weight and BMI Z scores but not in height Z score. In contrast, both urban as well as private school children demonstrated significant increases in height, weight and BMI Z scores. While the private and urban school children are becoming heavier and taller, the rural and government school children are simply becoming heavier and not taller. The findings also document that the time trend for linear growth remained stagnant in rural areas and low socioeconomic levels. It should be noticed that area of residence and type of school are acceptable surrogates of level of urbanization and socioeconomic status respectively in the Indian context. The government school children as well as the rural children are relatively poor. The private school children are relatively rich and the urban children are a mix of rich and poor. It is important to note that secular trends in height demonstrated during childhood could extend into adulthood [23]. This assumes significance due to the fact that adult height exhibits inverse linear associations with mortality from coronary heart disease and stroke as well as total mortality [24]. In addition, the study population exhibited relatively higher age, gender and height specific blood pressures suggesting that there is an increased cardiovascular risk visible even during childhood [25]. The combination of sub optimal adult height predictions with high blood pressures in childhood projects an adverse cardiovascular health profile in future for these children especially those belonging to low socio-economic levels.

All three sub group comparisons i.e. rural Vs urban, government Vs private as well as boys Vs girls demonstrated a significant widening of height difference. In terms of weight difference, significant decreases were seen in government Vs private as well as boys Vs girls comparisons. In terms of BMI difference, significant decrease was seen in government Vs private comparison. The study suggests that the socio economically advanced as well as the more urbanised segments of the pediatric population are growing relatively taller than their counterparts, promoting a progressive height divide in the population. The results also suggest that the weight divide between the higher and lower socio economic segments of the pediatric population is diminishing with time.

While boys have shown improvements in height, weight and BMI statuses, girls have shown improvements in weight and BMI statuses only. The gender divide in weight status is diminishing with time. Vidal et al reported a similar time trend of diminishing weight divide between the genders among children [22]. During the study period, boys showed an improvement in height status while girls showed a decline in the same as evidenced by their height Z scores at the end of the study. The enhancement of gender divide in terms of height seen in the present study appears to be in contrast to the trend demonstrated by Vidal et al [22]. This disparity in growth pattern between the genders could probably be due to the combined effects of both physiological and socio demographic influences.

It is evident that there is a shift in weight status as well as BMI status across all subgroups (Figures 1 &2). A notable difference between the two transitions is that the shift in BMI in the private school children appears less impressive than other sub groups. This is despite the fact that private school children too had significant shifts in weight status like other sub groups. The reason for this disparity could be the fact that private school children are growing more symmetrically and the change in BMI is less due to significant gains in height status that accompanies their weight shift. Combining the above findings, it becomes clear that the low socio-economic segments of the pediatric population are experiencing overweight issues that are growing at a faster rate when compared to the same in higher socio-economic segments. The asymmetry in the growth transition due to socio economic differences appears to be the major reason for this expansion of overweight population. The socio demographic segregation of weight and adiposity status distribution at the end of the study is expressed in Figure 3. The public health implications of this asymmetric, rapid growth transition in a population that already exhibits increased cardiovascular disease susceptibility could be serious.

The study indicates that the conversion of underweight to normal weight status occurs more in urban area and girls in comparison to rural area and boys respectively. The conversion of normal weight status to underweight status occurs more in rural area, government schools and boys when compared to their respective counterparts. The persistence of underweight appears to be significantly more with rural children and boys. These findings suggest that the favorable decline of underweight burden has socioeconomic and gender gradients.

During the study period (two years), the underweight population contracted by 22.2%. In the same period, the overweight sub population has grown by 30.8% and the obese sub population by 30.3%. A swift decline in the underweight population along with rapid growth of overweight and obese populations in the cohort suggests that the study population is going through an accelerated phase of nutritional transition. This transition is visible across the entire spectrum of weight distribution in the pediatric population.

The conversion of normal weight status to overweight status occurs more in urban areas, private schools, and boys in comparison to rural areas, government schools and girls respectively. In contrast, no significant influence of the same socio demographic factors was noticed in conversion of overweight population to normal weight status. In short, the beneficial conversion of overweight to normal weight appears to be due to other unidentified factors. The conversion of overweight to normal weight happening in the pediatric population could be due to multiple factors like individual awareness about overweight issues as well as attempts at the family, school, community, government or individual level to reduce the burden of overweight. The role of these factors as well as other unidentified ones need to be clearly identified for converting them into effective interventions aimed at reducing the burden of childhood obesity.

## Conclusion

The pediatric population is experiencing a rapid growth and nutritional transition. A decline in the underweight population coupled with an escalation of overweight population is visible in the study. The low socio economic subsets of the pediatric population appear to grow in an asymmetric, unhealthy pattern. The heterogeneous nature of this transition appears to be due to differences in socioeconomic levels as well as varying grades of urbanisation.

## Competing interests

The authors declare that they have no competing interests.

## Authors' contributions

MR conceived, designed and drafted the study. KRS did the statistical analysis and contributed to the drafting of manuscript. MP managed the data and assisted in drafting the manuscript. AS assisted in statistical analysis and in data management. RKK supervised and revised the manuscript for important intellectual content. All authors read and approved the final manuscript. MR will act as guarantor of the study.
